# Prevalence and Antimicrobial Susceptibility Patterns of Non-fermenting Gram-Negative Bacteria Causing Lower Respiratory Tract Infections: A Retrospective Study

**DOI:** 10.7759/cureus.88844

**Published:** 2025-07-27

**Authors:** Swadhin Choudhury, Basanti Kumari Pathi, Jyoti Prakash Sahoo, Shakya Mohanty, Kumudini Panigrahi

**Affiliations:** 1 Microbiology, Kalinga Institute of Medical Sciences, Bhubaneswar, IND; 2 Pharmacology, Kalinga Institute of Medical Sciences, Bhubaneswar, IND; 3 Critical Care Medicine, Kalinga Institute of Medical Sciences, Bhubaneswar, IND

**Keywords:** acinetobacter baumanii, antibiotics therapy, antimicrobial susceptibility pattern, bacterial drug resistance, burkholderia species, culture and sensitivity, lower respiratory tract infection (lrti), non-fermenters, non-fermenting gram-negative bacilli (nfgnb), pseudomonas infections

## Abstract

Background and objectives: Lower respiratory tract infections (LRTIs) increase the morbidity and hospital bed occupancy. Cases of LRTI due to non-fermenting Gram-negative bacilli (NFGNB) are common in healthcare institutions. The common NFGNB found nowadays are *Pseudomonas aeruginosa*, *Acinetobacter baumannii*, and *Burkholderia cepacia*. Prophylactic antibiotic use and frequent antimicrobial susceptibility testing (AST) impact the microbes' antimicrobial sensitivity patterns. We planned this study to estimate the prevalence of LRTI due to NFGNB and the antimicrobial sensitivity patterns of the causative microbes.

Methods: This retrospective study was conducted at Kalinga Institute of Medical Sciences (KIMS), Bhubaneswar, India. We analyzed data of the patients admitted between June 2023 and May 2025. We included female and male adult patients admitted to KIMS with LRTI during the study period. The specimens of bronchoalveolar lavage (BAL) fluid and endotracheal tube (ET) aspirate obtained from the qualified subjects were examined. Pathogenic bacteria were cultivated using enriched and selective media, such as 5% sheep blood agar and MacConkey agar. To identify isolates and evaluate AST, the VITEK 2 system (bioMérieux, Marcy l'Etoile, France) was employed. To analyze the data, we used R software (version 4.4.3; R Development Core Team, Vienna, Austria).

Results: Our hospital admitted 2,389 patients with LRTI during the research period. Of them, 757 (31.69%) patients had a positive culture result for NFGNB, and 362 (47.82%) participants were female. The study population's median age was 44.79 (38.11-66.43) years. We found three non-fermenters: *A. baumannii* (401, 52.97%), *P. aeruginosa* (248, 32.76%), and *B. cepacia* (108, 14.27%). The samples of *A. baumannii* and *B. cepacia *were highly sensitive to tigecycline. The cases with *P. aeruginosa* were mainly sensitive to aztreonam and demonstrated the least sensitivity to colistin.

Conclusion: The NFGNB encountered in this study were *A. baumannii*, *P. aeruginosa*, and *B. cepacia*. *P. aeruginosa* was highly sensitive to aztreonam. *B. cepacia* and *A. baumannii* had maximum sensitivity for tigecycline. Future studies with bigger sample sizes must examine the pathogenic NFGNB causing LRTI and their antimicrobial susceptibility patterns.

## Introduction

Lower respiratory tract infections (LRTIs) are among the most common global infectious diseases, causing substantial morbidity and mortality regardless of age [1]. LRTIs due to multidrug-resistant Gram-negative bacteria have become more widespread within the past 10 years [2,3]. Antibiotic therapies are started in many cases before the microbiological etiology is confirmed. Such infections, therefore, have a poorer prognosis than infections brought on by sensitive pathogens [2,4].

Non-fermenting gram-negative bacilli (NFGNB) commonly inhabit the soil, water, human skin, and gut. In healthcare facilities, they can be isolated from ventilators [5]. These bacteria are common sources of nosocomial infections. The risk of nosocomial infections is higher among patients admitted to the intensive care unit (ICU) and those with any malignancy or compromised immune systems [6]. In the Indian subcontinent, the most common NFGNB in various clinical samples are *Acinetobacter baumannii* and *Pseudomonas aeruginosa* [7-9]. *Stenotrophomonas maltophilia*, *Burkholderia cepacia*, and *Elizabethkingia meningoseptica* are other emerging NFGNB found in some patients [9,10].

Following initial exposure to NFGNB, persistent attachments to host cell receptors result in bacterial colonization [11,12]. Bacteria require the right environmental conditions (e.g., pH and oxygen) and nutrients (e.g., iron and amino acids) to proliferate after colonization. Under favorable conditions, exotoxins or extracellular enzymes are released. It harms the integrity of the mucosa and encourages immune system evasion. Eventually, it accelerates the local infection and systemic dissemination [11,13,14]. According to recent studies, NFGNB can facilitate its pathogenicity by secreting outer-membrane vesicles (OMVs), spherical outer membrane derivatives containing periplasmic material. OMVs produce virulence factors, weaken the host immune system, and produce biofilm [15,16].

Production of biofilm by many bacteria can shield them from environmental stress, dehydration, phagocytosis, and drugs [11,17]. It can exacerbate chronic respiratory disorders [18]. Hence, biofilm production is a significant risk factor for hospital-acquired infections [17-19]. Quorum sensing (QS) is a specialized communication system that depends on self-produced signaling molecules called autoinducers that function as hormone-like substances and regulate various metabolic activities [20].

Rising incidences of LRTIs due to NFGNB made us plan this study. We mapped this study to determine the prevalence of LRTIs due to NFGNB in our hospital and their antimicrobial susceptibility patterns.

## Materials and methods

Study design

This retrospective study was conducted at Kalinga Institute of Medical Sciences (KIMS), Bhubaneswar, India. We explored and analyzed the case sheets of the patients admitted to KIMS between June 2023 and May 2025 with LRTIs. Before conducting the study, we obtained ethical permission from the Institutional Ethics Committee (KIIT/KIMS/IEC/2253/2025 dated 31.05.2025).

Study participants

We included adult patients (of either gender) admitted to KIMS with LRTIs within the stipulated period. We excluded patients under 18 years, those with missing AST reports, or those referred from other hospitals with ongoing antibiotics. The patients with any findings other than NFGNB were also excluded.

Study procedure

The demographics, such as age and gender, and clinical parameters (e.g., presence of diabetes or hypertension and NFGNB isolates on the AST reports) of the recruited participants were noted. The endotracheal tube (ET) aspirate was obtained without bronchoscopy using a 14F, 22-inch suction catheter. The catheter was cautiously inserted 25-26 cm into the tube. Once the catheter was carefully aspirated without saline, it was removed from the tube. A sterile syringe was used to inject 3-4 mL of 0.9% saline to rinse the mucus collector. The sterile jar containing the ET aspirate had a screw-cap lid that was tightly sealed. A peripheral bronchiolar ramifications-connected fibrotic bronchoscope was used to provide 30-50 mL of normal saline to collect bronchoalveolar lavage (BAL) fluid. The saline was aspirated immediately and put in a sterile, dry, clean container.

Immediately after collection, respiratory samples were processed. If processing was anticipated to take longer than one to two hours, samples were stored at -80°C to -20°C to preserve the composition of the microbial population. An inoculating loop with a diameter of 2 mm was sterilized for smear fixing by heating it to red-hot and then letting it cool. A mucopurulent specimen was assessed by placing the loop flat on the liquid's surface. The loop of the slide was flattened in the middle and numbered. Before staining, the specimen was spread thinly on a glass slide, allowed to dry, and fixed with heat. The smear was outlined in a circle to improve visibility.

Selective and enriched media, such as 5% sheep blood agar and MacConkey agar, were utilized to cultivate NFGNB causing LRTIs. Semi-quantitative cultures were conducted using standard procedures using a nichrome wire loop and a calibrated loop method. Then, the plates were incubated aerobically for the entire night in an incubator with 5% CO₂. The plates were incubated for 24 and 48 hours before being checked for bacterial growth. For BAL fluid and ET aspirate, colony counts of > 10^4^ and > 10^5^ colony-forming units per millilitre (CFU/mL) were considered significant, respectively. Counts that fell short of these cutoffs were deemed irrelevant.

Initial identification was done using enzymatic tests such as catalase, coagulase, and oxidase. The Clinical and Laboratory Standards Institute (CLSI) 2022 cutoff levels were used to identify isolates and evaluate antibiotic susceptibility using the VITEK 2 system (bioMérieux, Marcy l'Etoile, France) [21]. A 64-well colorimetric reagent card was used in the VITEK 2 system. Using different cards, Gram-positive and Gram-negative microorganisms were distinguished. The organisms in the well were identified by their metabolic activities. The organism was identified with high confidence by comparing its response pattern to a database.

Aspartate aminotransferase (AST) operates via the principle of micro-broth dilution. After 18-24 hours, the microbe colonies were separated from the agar plates. Using saline solution or broth, those colonies were converted to suspension. A calibrated photometric device (bioMérieux, Marcy l'Etoile, France) controlled the suspension turbidity to 0.5 McFarland. The AST inoculum was injected into a 64-well colorimetric reagent card with a fixed antibiotic concentration. For this investigation, the AST card N406 was utilized. The minimum inhibitory concentration (MIC) values were interpreted. The Advanced Expert System (AES) corroborated the final MIC result before providing the antimicrobial susceptibility.

Statistical analysis

Convenience sampling was used to conduct this retrospective study. The normality of the data distribution was gauged with the Shapiro-Wilk test. Median and interquartile range (IQR) were used to express the continuous data. The categorical data were shown as frequency and proportion. We used chord diagrams to portray pathogenic bacteria's AST results (i.e., resistant, intermediate, or sensitive). R software (version 4.4.3; R Development Core Team, Vienna, Austria) was used to compute the data [22]. Statistical significance was regarded as a p-value of 0.05 or less.

## Results

During the study period, 2,389 patients were admitted to our hospital with LRTIs. Of them, 161 (6.73%) had been referred with some ongoing antimicrobials. Gram-positive cocci and *Enterobacterales *were found in 144 (6.03%) and 1,287 (53.87%) patients, respectively. Eighteen patients had double microbial sources of infection. Twenty-two case sheets were deficient in the culture and sensitivity reports. The remaining 757 (31.69%) patients with a positive culture report for NFGNB were considered for analysis. Table 1 narrates the sociodemographic and clinical characteristics of the study participants. A total of 362 (47.82%) of 757 participants were women. The study population's median age was 44.79 (38.11-66.43) years. The non-fermenters encountered in our study were *A. baumannii* (401, 52.97%), *P. aeruginosa* (248, 32.76%), and *B. cepacia* (108, 14.27%).

**Table 1:** Demographic and clinical characteristics of the study population The median and interquartile range (IQR) were used to depict the continuous variables. Frequency and percentage were used to display the category variables.

Parameters	Value
Total participants	757
Age (in years)	44.79 (38.11-66.43)
Female	362 (47.82%)
Comorbidities	
Type 2 diabetes mellitus	98 (54.14%)
Hypertension	117 (64.64%)
NFGNB isolates	
Acinetobacter baumannii	401 (52.97%)
Pseudomonas aeruginosa	248 (32.76%)
Burkholderia cepacia	108 (14.27%)

**Figure 1:**
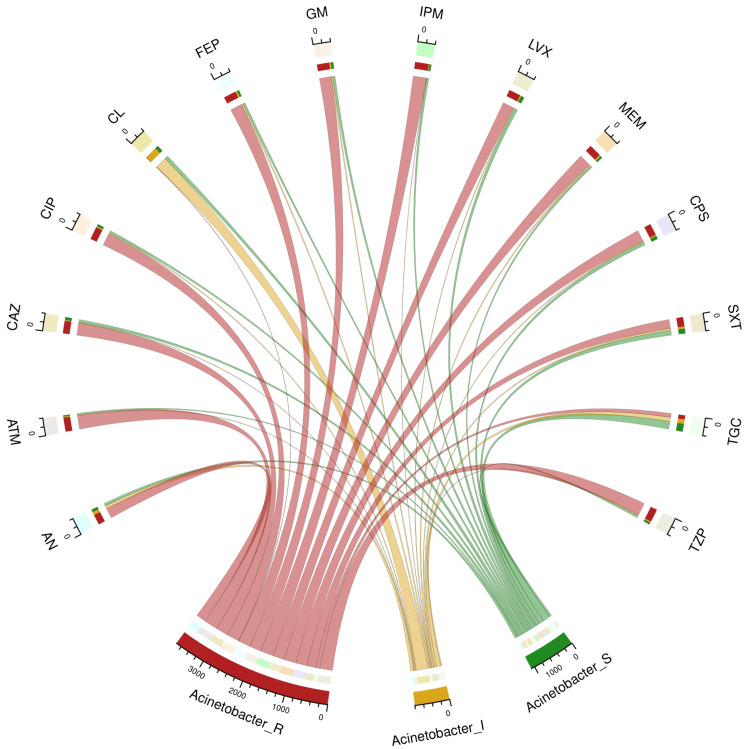
Antimicrobial sensitivity patterns of 401 isolates of Acinetobacter baumannii The upper and lower portions of the chord diagram demonstrate the 14 antibiotics used in AST and the antibiotic susceptibility patterns (R: resistant, I: intermediate, and S: sensitive) of *Acinetobacter baumannii*, respectively. The bandwidths correspond to the antimicrobial susceptibility of *Acinetobacter baumannii to* the 14 different drugs. AN: amikacin, ATM: aztreonam, CAZ: ceftazidime, CIP: ciprofloxacin, CL: colistin, FEP: cefepime, GM: gentamicin, IPM: imipenem, LVX: levofloxacin, MEM: meropenem, CPS: cefoperazone-sulbactam, SXT: cotrimoxazole, TGC: tigecycline, TZP: piperacillin-tazobactam, AST: antimicrobial susceptibility testing.

Table 2 and Figure 1 illustrate the antimicrobial sensitivity pattern of 401 specimens of *A. baumannii* found in the study population. The sensitivity was the highest towards tigecycline (195, 48.6%), followed by cotrimoxazole (121, 30.1%), cefoperazone-sulbactam (88, 21.9%), and colistin (87, 21.7%). These isolates demonstrated their maximum resistance to aztreonam (345, 86.0%), followed by imipenem (335, 83.5%), piperacillin-tazobactam (324, 80.8%), ciprofloxacin (317, 79.1%), meropenem (312, 77.8%), and ceftazidime (311, 77.6%).

**Table 2:** Antibiotic susceptibility patterns of non-fermenters Antimicrobial susceptibility patterns of NFGNB are illustrated as frequency and proportions AN: amikacin, ATM: aztreonam, CAZ: ceftazidime, CIP: ciprofloxacin, CL: colistin, FEP: cefepime, GM: gentamicin, IPM: imipenem, LVX: levofloxacin, MEM: meropenem, CPS: cefoperazone-sulbactam, SXT: cotrimoxazole, TGC: tigecycline, TZP: piperacillin-tazobactam, NFGNB: non-fermenting Gram-negative bacteria

Bacteria	AN	ATM	CAZ	CIP	CL	FEP	GM	IPM	LVX	MEM	CPS	SXT	TGC	TZP
*Acinetobacter baumannii* (n = 401)														
Sensitive	68 (17.0%)	34 (8.5%)	73 (18.2%)	57 (14.2%)	87 (21.7%)	54 (13.5%)	64 (16.0%)	54 (13.5%)	84 (20.9%)	53 (13.2%)	88 (21.9%)	121 (30.1%)	195 (48.6%)	50 (12.5%)
Intermediate	77 (19.2%)	22 (5.5%)	17 (4.2%)	27 (6.7%)	302 (75.3%)	39 (9.7%)	34 (8.4%)	12 (3.0%)	26 (6.5%)	36 (9.0%)	26 (6.5%)	50 (12.5%)	106 (26.4%)	27 (6.7%)
Resistant	256 (63.8%)	345 (86.0%)	311 (77.6%)	317 (79.1%)	12 (3.0%)	308 (76.8%)	303 (75.6%)	335 (83.5%)	291 (72.6%)	312 (77.8%)	287 (71.6%)	230 (57.4%)	100 (25.0%)	324 (80.8%)
*Pseudomonas aeruginosa* (n = 248)														
Sensitive	169 (68.2%)	192 (77.4%)	129 (52.0%)	104 (41.9%)	103 (41.5%)	144 (58.1%)	108 (43.5%)	128 (51.6%)	85 (34.3%)	95 (38.3%)	106 (42.7%)	15 (6.1%)	95 (38.3%)	120 (48.4%)
Intermediate	10 (4.0%)	25 (10.1%)	24 (9.7%)	51 (20.6%)	130 (52.4%)	38 (15.3%)	61 (24.6%)	37 (14.9%)	73 (29.4%)	57 (23.0%)	57 (23.0%)	48 (19.3%)	35 (14.1%)	44 (17.7%)
Resistant	69 (27.8%)	31 (12.5%)	95 (38.3%)	93 (37.5%)	15 (6.1%)	66 (26.6%)	79 (31.9%)	83 (33.5%)	90 (36.3%)	96 (38.7%)	85 (34.3%)	185 (74.6%)	118 (47.6%)	84 (33.9%)
*Burkholderia cepacia* (n = 108)														
Sensitive	28 (25.9%)	45 (41.7%)	49 (45.4%)	34 (31.4%)	0	38 (35.2%)	24 (22.2%)	41 (38.0%)	12 (11.1%)	36 (33.3%)	50 (46.3%)	6 (5.5%)	60 (55.6%)	27 (25.0%)
Intermediate	13 (12.0%)	35 (32.4%)	23 (21.3%)	37 (34.3%)	7 (6.5%)	44 (40.7%)	51 (47.2%)	32 (29.6%)	17 (15.7%)	37 (34.3%)	36 (33.3%)	38 (35.2%)	27 (25.0%)	34 (31.5%)
Resistant	67 (62.1%)	28 (25.9%)	36 (33.3%)	37 (34.3%)	101 (93.5%)	26 (24.1%)	33 (30.6%)	35 (32.4%)	79 (73.2%)	35 (32.4%)	22 (20.4%)	64 (59.3%)	21 (19.4%)	47 (43.5%)

**Figure 2:**
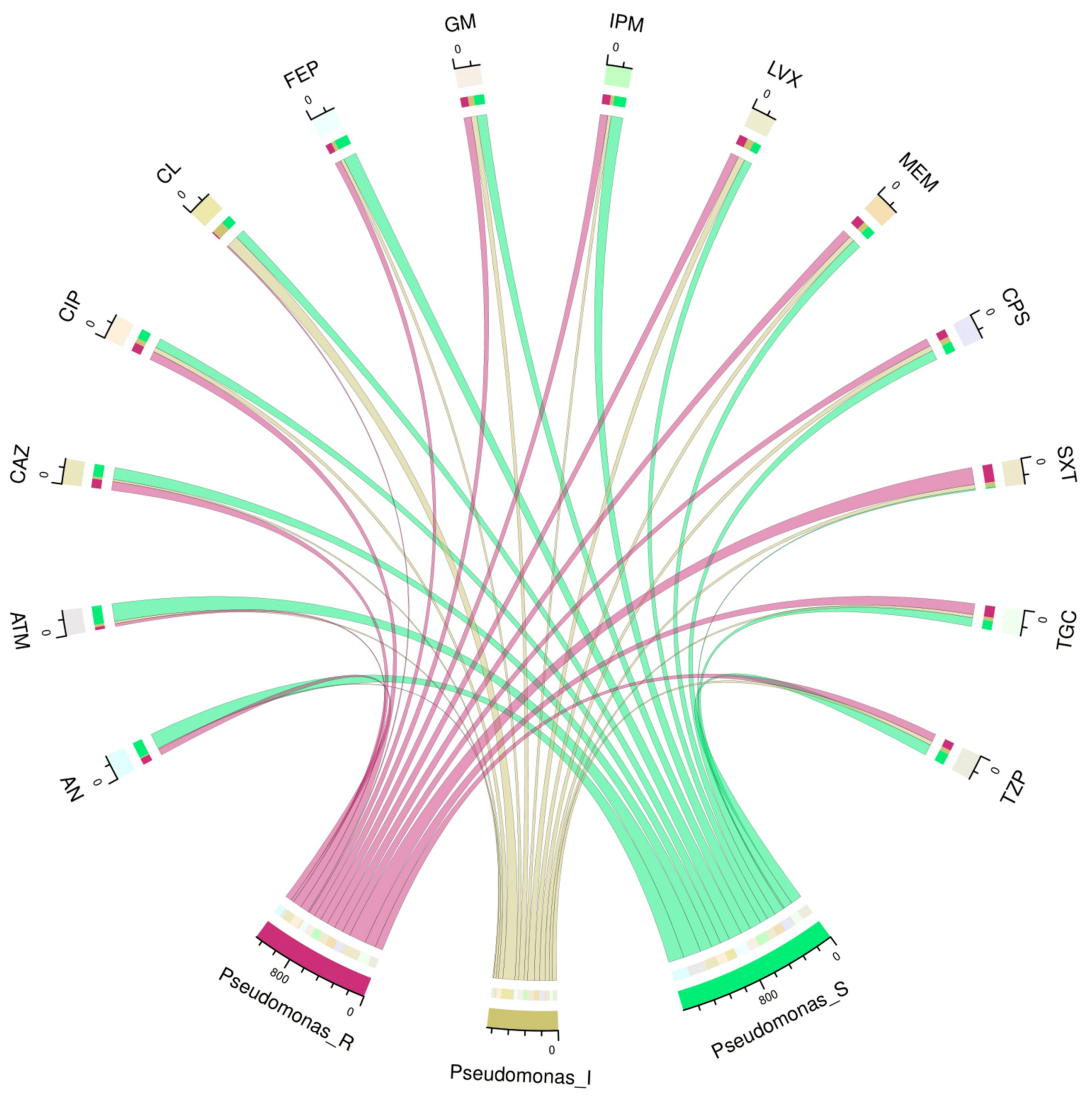
Antimicrobial sensitivity patterns of 248 isolates of Pseudomonas aeruginosa The upper and lower portions of the chord diagram demonstrate the 14 antibiotics used in AST and the antibiotic susceptibility patterns (R: resistant, I: intermediate, and S: sensitive) of *Pseudomonas aeruginosa*, respectively. The bandwidths correspond to the antimicrobial susceptibility of *Acinetobacter baumannii* to the 14 different drugs AN: amikacin, ATM: aztreonam, CAZ: ceftazidime, CIP: ciprofloxacin, CL: colistin, FEP: cefepime, GM: gentamicin, IPM: imipenem, LVX: levofloxacin, MEM: meropenem, CPS: cefoperazone-sulbactam, SXT: cotrimoxazole, TGC: tigecycline, TZP: piperacillin-tazobactam, AST: antimicrobial susceptibility testing

Table 2 and Figure 2 illustrate the antimicrobial sensitivity pattern of 248 specimens of *Pseudomonas aeruginosa* found in the study population. The sensitivity was the highest towards aztreonam (192, 77.4%), followed by amikacin (169, 68.2%), cefepime (144, 58.1%), ceftazidime (129, 52.0%), and imipenem (128, 51.6%). These isolates demonstrated their maximum resistance to cotrimoxazole (185, 74.6%), followed by tigecycline (118, 47.6%), meropenem (96, 38.7%), ceftazidime (95, 38.3%), and ciprofloxacin (93, 37.5%).

**Figure 3:**
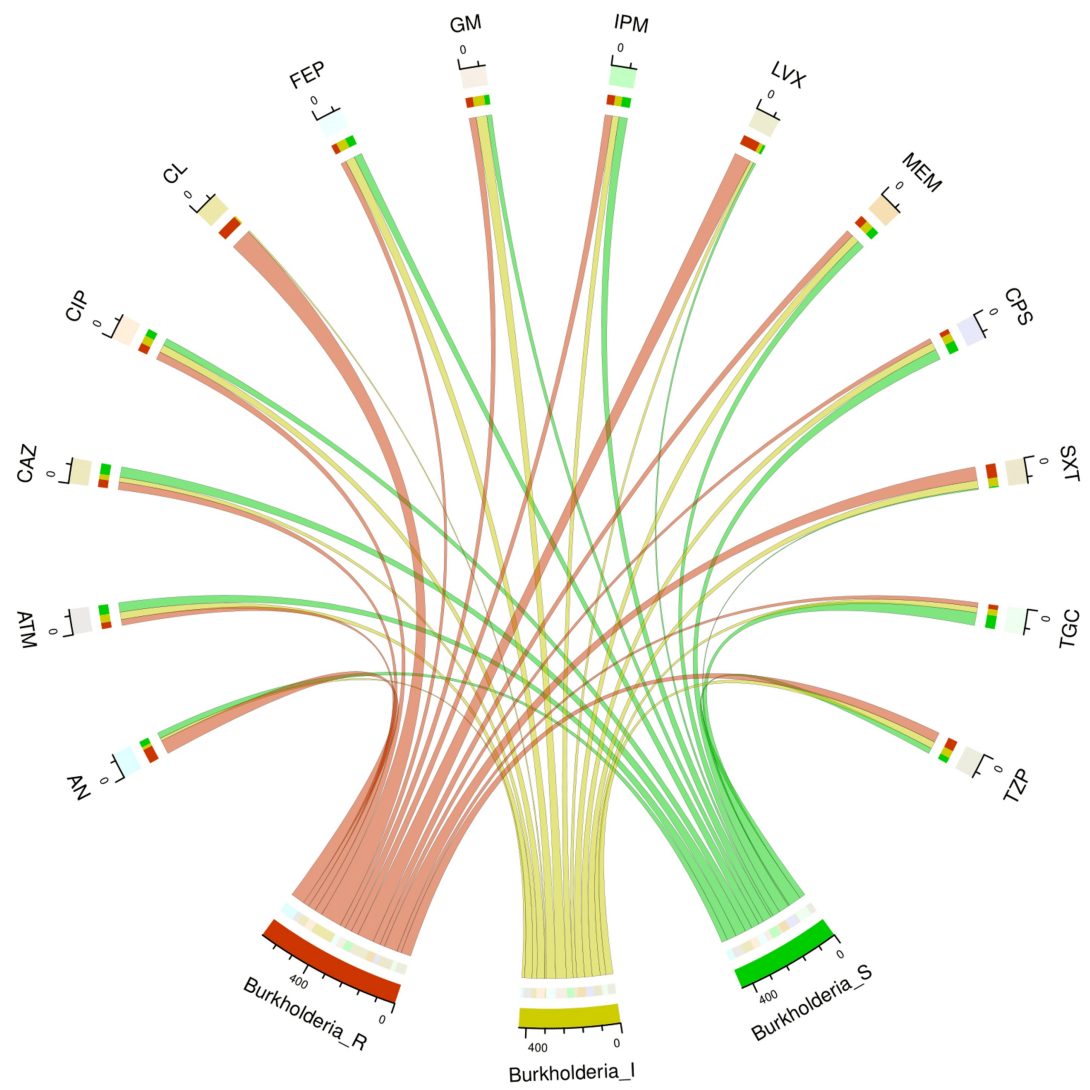
Antimicrobial sensitivity patterns of 108 isolates of Burkholderia cepacia The upper and lower portions of the chord diagram demonstrate the 14 antibiotics used in AST and the antibiotic susceptibility patterns (R: resistant, I: intermediate, and S: sensitive) of *Burkholderia cepacia*, respectively. The bandwidths correspond to the antimicrobial susceptibility of *Acinetobacter baumannii* to the 14 different drugs AN: amikacin, ATM: aztreonam, CAZ: ceftazidime, CIP: ciprofloxacin, CL: colistin, FEP: cefepime, GM: gentamicin, IPM: imipenem, LVX: levofloxacin, MEM: meropenem, CPS: cefoperazone-sulbactam, SXT: cotrimoxazole, TGC: tigecycline, TZP: piperacillin-tazobactam, AST: antimicrobial susceptibility testing

Table 2 and Figure 3 illustrate the antimicrobial sensitivity pattern of 108 specimens of *B. cepacia* found in the study population. The sensitivity was the highest towards tigecycline (60, 55.6%), followed by cefoperazone-sulbactam (50, 46.3%), ceftazidime (49, 45.4%), aztreonam (45, 41.7%), and imipenem (41, 38.0%). None of the *B. cepacia *isolates demonstrated sensitivity to colistin. These isolates demonstrated their maximum resistance to colistin (101, 93.5%), followed by levofloxacin (79, 73.2%), amikacin (67, 62.1%), cotrimoxazole (64, 59.3%), and piperacillin-tazobactam (47, 43.5%).

## Discussion

In this retrospective study, 757 (31.69%) of 2,389 patients admitted with LRTIs had positive culture reports for NFGNB. Of those 757 participants, 362 (47.82%) were women. The study population's median age was 44.79 (38.11-66.43) years. In our investigation, we found three non-fermenters (in ascending order): *B. cepacia* (108, 14.27%), *P. aeruginosa* (248, 32.76%), and *A. baumannii* (401, 52.97%). Our observations concurred with the findings of recent studies [23-25]. Dash et al. [23] conducted a five-year (2019-2023) trend analysis of antimicrobial susceptibility patterns of NFGNB in our hospital. Our results differ from theirs in that we found more cases of NFGNB overall and in females.

*A. baumannii*, *P. aeruginosa*, and *B. cepacia* are among the NFGNB types associated with increasing drug resistance [7-9]. The bacteria may have built-in resistance mechanisms, change their genes through plasmids or mutations, or acquire drug-resistance genes [11-20]. Among the various resistance mechanisms are changes in membrane permeability, target site modifications, efflux pumps, porin channels, and the synthesis of enzymes that render drugs inactive [11,23]. Growing antimicrobial resistance among GNB makes empirical therapy problematic because only a few antimicrobial drugs are effective in controlling infections caused by these resistant organisms, which has made antimicrobial resistance a significant challenge in the field of global public health [24,25]. Since each nation and region has its own pattern of organism resistance, monitoring local resistant patterns can help direct the prudent use of antimicrobial agents and control antimicrobial resistance [25].

The sensitivity of *A. baumannii* to tigecycline was the highest (195, 48.6%). Maximum resistance was found towards aztreonam (345, 86.0%), followed by imipenem (335, 83.5%), piperacillin-tazobactam (324, 80.8%), ciprofloxacin (317, 79.1%), meropenem (312, 77.8%), and ceftazidime (311, 77.6%). Aztreonam (192, 77.4%) had the highest sensitivity for *P. aeruginosa*. These isolates exhibited the highest level of resistance to ceftazidime (95, 38.3%), ciprofloxacin (93, 37.5%), tigecycline (118, 47.6%), meropenem (96, 38.7%), and cotrimoxazole (185, 74.6%). Tigecycline exhibited the highest sensitivity (60, 55.6%) against *B. cepacia*. These isolates did not show any signs of colistin sensitivity. Widespread usage of colistin against *B. cepacia* could have made it naturally resistant to colistin [26]. The isolates exhibited the highest levels of resistance to colistin (101, 93.5%) and levofloxacin (79, 73.2%). Baruah et al. [27] found *B. cepacia* to be the most common NFGNB in their study. Goel et al. [28] mentioned *P. aeruginosa* as the most prevalent NFGNB in their study. Our study findings differ from both of the above studies. This difference could be attributed to the heterogeneity of the study population, the antimicrobial policy of the concerned hospital, and the virulence of the microbes in different regions and times.

The use of chord diagrams to illustrate the data was this study's primary strength. Additionally, our study has certain limitations. First, the history of recent illnesses and antibiotic use was not examined. Second, we did not examine the relationship between the AST results, hospitalization, and outcome. Third, there are several risk factors and etiologies for pneumonia in ICU patients. We did not evaluate any risk variables for LRTI development. Fourth, we also did not evaluate patient outcome data such as mortality, duration of ICU stay, and complications.

## Conclusions

*A. baumannii*, *P. aeruginosa*, and *B. cepacia* were our hospital's most prevalent NFGNB causing LRTIs. *A. baumannii* and *B. cepacia* were highly sensitive to tigecycline. Aztreonam sensitivity was the highest in *P. aeruginosa*. We recommend more prospective studies with heterogeneous populations to determine the incidence of NFGNB-induced infections and their antimicrobial susceptibility patterns.
